# Application of Network Scale Up Method in the Estimation of Population Size for Men Who Have Sex with Men in Shanghai, China

**DOI:** 10.1371/journal.pone.0143118

**Published:** 2015-11-18

**Authors:** Jun Wang, Ying Yang, Wan Zhao, Hualin Su, Yanping Zhao, Yue Chen, Tao Zhang, Tiejun Zhang

**Affiliations:** 1 Department of Epidemiology, School of Public Health, Fudan University, Shanghai, China; 2 Key Laboratory of Public Health Safety (Fudan University), Ministry of Education, Shanghai, China; 3 Minhang district center for diseases control and prevention, Shanghai, China; 4 School of Epidemiology, Public Health and Preventive Medicine, University of Ottawa, Ontario, Canada; Centers for Disease Control and Prevention, UNITED STATES

## Abstract

**Background:**

Men who have sex with men (MSM) are at high risk of HIV infection. For developing proper interventions, it is important to know the size of MSM population. However, size estimation of MSM populations is still a significant public health challenge due to high cost, hard to reach and stigma associated with the population.

**Objectives:**

We aimed to estimate the social network size (*c* value) in general population and the size of MSM population in Shanghai, China by using the net work scale-up method.

**Methods:**

A multistage random sampling was used to recruit participants aged from 18 to 60 years who had lived in Shanghai for at least 6 months. The “known population method” with adjustment of backward estimation and regression model was applied to estimate the *c* value. And the MSM population size was further estimated using an adjusted *c* value taking into account for the transmission effect through social respect level towards MSM.

**Results:**

A total of 4017 participants were contacted for an interview, and 3907 participants met the inclusion criterion. The social network size (*c* value) of participants was 236 after adjustment. The estimated size of MSM was 36354 (95% CI: 28489–44219) for the male Shanghaies aged 18 to 60 years, and the proportion of MSM among the total male population aged 18 to 60 years in Shanghai was 0.28%.

**Conclusions:**

We employed the network scale-up method and used a wide range of data sources to estimate the size of MSM population in Shanghai, which is useful for HIV prevention and intervention among the target population.

## Introduction

Size estimates of key populations at high-risk for HIV infection, including female sex workers (FSW), inject drugs users (IDU) and men who have sex with men (MSM), are needed to better understand HIV epidemics and plan appropriate interventions and allocate sufficient resources [1–4]. It has been made a priority by the World Health Organization (WHO) and the Joint United Nations Programme on HIV/AIDS (UNAIDS) [5–7]. By estimating the size of populations at high risk of HIV, a country can revise its strategic plans and resource programmes appropriately, improve modelling of its epidemic, and advocate for services for those populations purposely [8].

Men who have sex with men (MSM) are disproportionately vulnerable to HIV/AIDS throughout the world [9, 10]. Previous epidemiological data have shown that MSM have become one of the most important populations in the fight against HIV/AIDS, and the concentrated epidemics prevail among this population in much of the world [11]. The HIV transmission among MSM has also been a challenge in China [12]. Despite the epidemiological evidence available, MSM appears to be a neglected group, and data on the MSM population size are sparse and inconsistent [13, 14]. This would in turn hinder the HIV prevention and intervention among this high-risk group. Moreover, China is a big country with diverse social culture background across different geographic regions, which could cause different homosexual subculture. It is important to estimate this specific population at high risk for HIV infection. However, because of the stigma against high-risk populations for HIV/AIDS, MSM became very hard to contact [15], which makes considerable difficulty in getting an accurate estimate for this population in China. It is of urgency to explore a suitable method to better estimate the population size.

Currently, there are several methods being adapted to estimate the size of HIV high-risk populations, including multiplier, nomination methods and capture-recapture etc. [16–18]. However, traditional methods such as multiplier and capture-recapture were not accurate enough because these methods require direct contact with hard-to-reach populations. A relatively new method, the network scale-up method (NSUM), was initially proposed after the Mexico City earthquake in 1989 [19]. This is a population-based survey method and does not need to directly contact high-risk populations. The method can also estimate the size of multiple populations in a single survey [4]. It has been proved to be a promising and apparently simple and inexpensive population size estimation technique for HIV high-risk populations [20]. Given the absence of a globally accepted gold standard for key population size estimation, we employed the network scale-up method to estimate the size of MSM in Shanghai, China. Results from the present study could benefit the HIV prevention among MSM in China and some other countries sharing similar situation.

## Material and Methods

### Study sites

A community-based study was conducted in Minhang district, one of the 19 districts in Shanghai, China, between September and December 2014. Shanghai is one of the most populous and economically developed metropolises in China, with 24 million registered residents. The first HIV case was reported in Shanghai in the year of 1987. Recently, surveillance data in Shanghai have also shown that MSM are at high risk of HIV infection (Shanghai Center for Disease Prevention and Control, 2014).

### Study participants

To be eligible for the present survey, a study participant had to be a person who; 1) was between 18 to 60 years of age; 2) had lived in Shanghai for at least 6 months; 3) had no any physician-diagnosed psychological problems; and 4) provided a written consent for participating in this study.

### Procedures

A multi-stage random cluster sampling was used to recruit participants. In the first stage, Minhang district was randomly selected from 19 districts in Shanghai. Within the selected district, there are a total of 57 communities were administratively included. At the second stage, 30 out of the 57 enumeration communities were randomly selected. Then a bibliographic list of neighborhoods from selected communities was obtained, and 170 neighborhoods were then drawn from the chosen communities. Overall, 4017 households were randomly selected, and from each household one person was randomly chosen from all eligible persons in each household for the survey.

### Network scale up method

Bernard et al. originated the network scale up method [21, 22], which is based on the assumption that participants’ personal social networks reflect the general population in a given region. This method assumes a total population T of size t and a subpopulation E of T with size e. The basic assumption can be formulized as follows:

$$\frac{m}{c}=\frac{e}{t}$$ (1)

Where *m* is the mean number of people known in subpopulation *E* of size e and *c* is the mean social network size of the people in total population *T* of size *t*. A maximum likelihood estimator [23], which based on that *i* participant knew *m*_*i*_ MSM follows a Binomial distribution:

$$\text{Probability }(m_{i})=\left(\begin{array}{c} C_{i}\\ m_{i} \end{array}\right)p^{m_{i}}{\left(1-p\right)}^{c_{i}-m_{i}}\;p=e_{j}/t.$$ (2)

Where *c*_*i*_ is the social network size of the survey participants, *e*_*j*_ is the population size of subgroup *j*. The *e* estimation of subpopulation *E* size is given by

$$ê=t\cdot \frac{\sum_{i=1}^{t}m_{ij}}{\sum_{i=1}^{t}c_{i}}$$ (3)

which has been testified as an unbiased formula. Where *m*_*ij*_ is the number of people in subgroup *j* (totally L subgroups which the size were known) that the survey participant *i* knows. And the estimating of *e* requires estimating the social network size *ci*. In the study, the “known population method” [21] was applied to estimate the *ci* value:

$${\hat{c}}_{i}=\left(\frac{\sum_{j=1}^{L}m_{ij}}{\sum_{j=1}^{L}e_{j}}\right)\cdot t$$ (4)

### C value estimation and adjustment

The “known population method” was used in the study to estimate the value of average social network size (*c* value). Backward estimation and regression model were applied to adjust the *c* value. Backward estimation was conducted by assuming one “known population” was missing, then using the average *c* value generated by the rest of the other known populations to calculate the estimated population size of each subpopulation. The ratio between the estimated size and the real statistic size out of the range 0.5–2.0 indicated that the estimate of the known population was underestimated or overestimated; therefore such populations were unsuitable for further analysis and excluded. Regression model which we set up with the mean number known in each subpopulation as dependent variable and the relative size of the subpopulations as independent variables, followed with a graphical analysis of residuals to kick out the abnormal populations. Finally the unsuitable populations were excluded by combining both the graphical analysis of residuals and backward estimation.

### Estimation of MSM population size with adjustment of social respect level

The estimation of MSM population size was calculated using formula (3). A transmission effect arises when a respondent does not count his/her contact as being in the group of interest, for example the respondent does not know that the contact belongs to the group. This bias can be large when a group is stigmatized. An adjustment was made in the current study considering the social desirable bias. Finally the participants were asked about their attitude towards MSM on a scale of five grades ranking from 1 to 5 points, and MSM population size was further adjusted based on their attitudes. In brief, similar to the study of Ukraine [24], the participants in our study were asked to rank their respect for MSM on a scale with 1 = very low to 5 = very high, among which 2, 3, 4 represent a respect level of low, medium or high. In the present study, the number of MSM that a participant knew was weighted with a factor of *W*_*i*_, which was used to reflect the impact of respect level on knowing MSM among interviewees. *W*_*i*_ was defined as the average number of MSM known to participants with a given respect level divided by the average number of MSM known to the participants with a medium level of respect (rank scale = 3 and shown as *M*_*3*_), and calculated by the formula of *W*_*i*_ = *M*_*i*_/*M*_*3*_. With introduction of the factor *W*_*i*_, Eq 3 was transformed into Eq 5 to estimate MSM population size.

$$ê=\frac{t}{\sum_{i=1}^{t}c_{i}}\cdot \sum_{i=1}^{t}m_{ij}\cdot W_{i}$$ (5)

All the known population sizes were obtained from the 2014 Shanghai Statistical Yearbook, Shanghai Bureau of Justice. Detailed information was described in Table 1.

**Table 1:** The official figure and estimated size of selected known populations after back estimation comparison of Shanghai.

Known population	Real data	Estimated data	Ratio of estimated to real	Data source	Include or exclude
Males aged 20–30	1019896	2230732	2.19	2014 Statistical Yearbook	exclude
Males aged 70 or older	734187	808592	1.10	2014 Statistical Yearbook	include
Females aged 20–30	989929	2160496	2.18	2014 Statistical Yearbook	exclude
Females aged 70 or older	938122	796442	0.85	2014 Statistical Yearbook	include
Junior high school students in 2013	436700	374488	0.86	2014 Statistical Yearbook	include
Senior high school students in 2013	156800	392850	2.51	2014 Statistical Yearbook	exclude
Men who divorced in 2013	139100	73630	0.53	2014 Statistical Yearbook	include
Men who married in 2013	221700	276652	1.24	2014 Statistical Yearbook	include
People who went to jail in 2013	6300	12044	1.90	Shanghai Bureau of Justice	include
People who logopathy	4015	28166	7.02	2014 Statistical Yearbook	exclude
People who are foreigners	176363	69293	0.39	2014 Statistical Yearbook	exclude
People who are doctors	58070	275900	4.75	2014 Statistical Yearbook	exclude
People who are lawyers	15189	55430	3.65	2014 Statistical Yearbook	exclude
People who died in 2013	116700	91994	0.79	2014 Statistical Yearbook	include
People who gave birth in 2013	108900	224765	2.06	2014 Statistical Yearbook	exclude
People who went abroad in 2013	116600	56757	0.49	2014 Statistical Yearbook	exclude
People who participated in Retail	879300	172852	0.09	2014 Statistical Yearbook	exclude
People who participated in commercial insurance	256400	317264	1.24	2014 Statistical Yearbook	include
People who had an accident in 2013	2011	28172	14.01	2014 Statistical Yearbook	exclude
People who died in traffic in 2013	914	13057	14.29	2014 Statistical Yearbook	exclude
People who volunteered blood donation in 2013	470500	135661	0.29	2014 Statistical Yearbook	exclude
People who retired in 2013	3906300	82166	0.02	2014 Statistical Yearbook	exclude

### Questionnaire interview

An anonymous and face-to-face questionnaire interview was administered to all the participants. The questionnaire included information on participants’ socio-demographic characteristics, social network (number of people they knew in 22 subgroups with known population size and number of MSM they knew), and personal attitude towards high-risk populations for HIV/AIDS, MSM in particular. The completed anonymous questionnaires were placed in a large box containing other completed questionnaires, reassuring each participant that no one could identify their completed questionnaire. All interviews took place in a private location. A small incentive equivalent to U.S. $5 was given to each participant.

In the present study, the participants were asked how many people they knew in the subpopulations with known size and how many MSM they knew. The working definition of “people they knew” was a person who: 1) the study participant had met in person before; 2) the participant knows by sight or name; 3) the participant had contacted within the last 2 years via phone calls or emails; and 4) had lived in Shanghai for at least 6 months.

### Statistical analysis

The original data were entered and managed in EpiData3.1 (The EpiData Association, Odense, Denmark). All data subsequently transferred to an SPSS database for further statistical analysis. Demographic characteristics were analyzed using descriptive statistics, i.e., mean, median and interquartile range (IQR) for continuous variables, and frequencies and proportions for categorical variables. Analyses were performed in SPSS version 18.0 (Version 18.0. Chicago: SPSS Inc.). All statistical tests were two sides, and the results were considered significant at the ɑ level of 0.05.

### Ethics Statement

This study was approved by the Institutional Review Board of Fudan University, China. Written consent was obtained before any procedures were performed for all the participants.

## Results

### Socio-demographic characteristics of the study participants

Of 4017 participants, 110 were excluded because their age was either beyond 60 or below 18. The remaining 3907 participants included 1920 men (49.1%), and had an average age of 40.54 (SD = 11.69) years. Other socio-demographic characteristics are detailed in Table 2. Most study participants (97.1%) were of Han ethnicity, the major ethnic group in China. Approximately 43.1% participants had received junior college or higher education, and the majority of them (78.7%) were married. Most participants (63.8%) had lived in Shanghai for more than 10 years, and the median residence time was 26.0 years (IQR: 7.00–46.00).

**Table 2:** Socio-demographic characteristics and average social network size of study participants.

	Sampling size	Percent(%)	Crude *c* value	95% CI
**Gender**				
Male	1920	49.1	248	238.07–258.56
Female	1987	50.9	234	224.53–242.79
**Age (years)**				
18–25	439	11.2	242	223.76–261.20
26–35	1160	29.7	242	229.60–254.57
36–45	835	21.4	247	231.22–261.90
46–55	977	25.0	233	219.18–246.46
56–60	496	12.7	243	222.72–262.88
**Education**				
Illiteracy	40	1.0	330	241.71–418.29
Primary school	266	6.8	190	169.23–210.43
Junior high	745	19.1	203	190.78–215.99
Senior high	1173	30.0	240	226.86–252.39
College or above	1683	43.1	264	253.08–275.38
**Marital status** *				
Single	562	14.4	256	236.90–275.81
Married	3074	78.7	237	229.68–244.73
Cohabited	82	2.1	215	158.53–272.05
Divorced	145	3.7	250	213.60–287.12
Widowed	41	1.0	312	229.16–396.00
**Residence**				
6 months -12 months	150	3.8	175	149.51–199.48
1–10 years	1263	32.3	221	209.88–231.93
more than 10 years	2494	63.8	255	245.88–263.86
**Career**				
Worker	1021	26.1	214	201.55–225.61
Administrative staff	528	13.5	288	266.43–309.27
Specialized Personnel	789	20.2	278	260.66–294.35
Service	354	9.1	246	223.25–268.90
Others	1215	31.1	218	206.72–229.03

* With missing data

### Crude estimation of social network size (c value)

Based on the formula used in the present study, the crude average social network size (*c* value) among participants was estimated at 241 (95% CI: 234–248). Since the *c* value is very important for improving the estimation accuracy, we combined regression model with backward estimation to adjust the *c* value.

### Adjustment with backward estimation and regression model

To identify appropriate subpopulations with known size to be used in the present study, the ratio between estimated size and real statistic size were calculated. Initially, a total of 22 subpopulations were included in the analysis. By using the criteria of exclusion ratio beyond the range of 0.5–2.0, 14 subpopulations were then excluded (Table 3).

**Table 3:** The known populations included or excluded combined with graphical analysis of residuals and backward estimation.

Known population	Regression model	Backward estimation	Combo
Males aged 20–30	exclude	exclude	exclude
Males aged 70 or older	exclude	include	exclude
Females aged 20–30	exclude	exclude	exclude
Females aged 70 or older	exclude	include	exclude
Junior high school students in 2013	include	include	include
Senior high school students in 2013	exclude	exclude	exclude
Men who divorced in 2013	include	include	include
Men who married in 2013	include	include	include
People who went to jail in 2013	include	include	include
People who logopathy	include	exclude	exclude
People who are foreigners	include	exclude	exclude
People who are doctors	exclude	exclude	exclude
People who are lawyers	include	exclude	exclude
people who died in 2013	include	include	include
People who gave birth in 2013	include	exclude	exclude
People who went abroad in 2013	include	exclude	exclude
People who participated in Retail	exclude	exclude	exclude
People who participated in commercial insurance	include	include	include
People who had an accident in 2013	include	exclude	exclude
People who died in traffic in 2013	include	exclude	exclude
People who volunteered blood donation in 2013	exclude	exclude	exclude
People who retired in 2013	exclude	exclude	exclude

**Fig 1:**
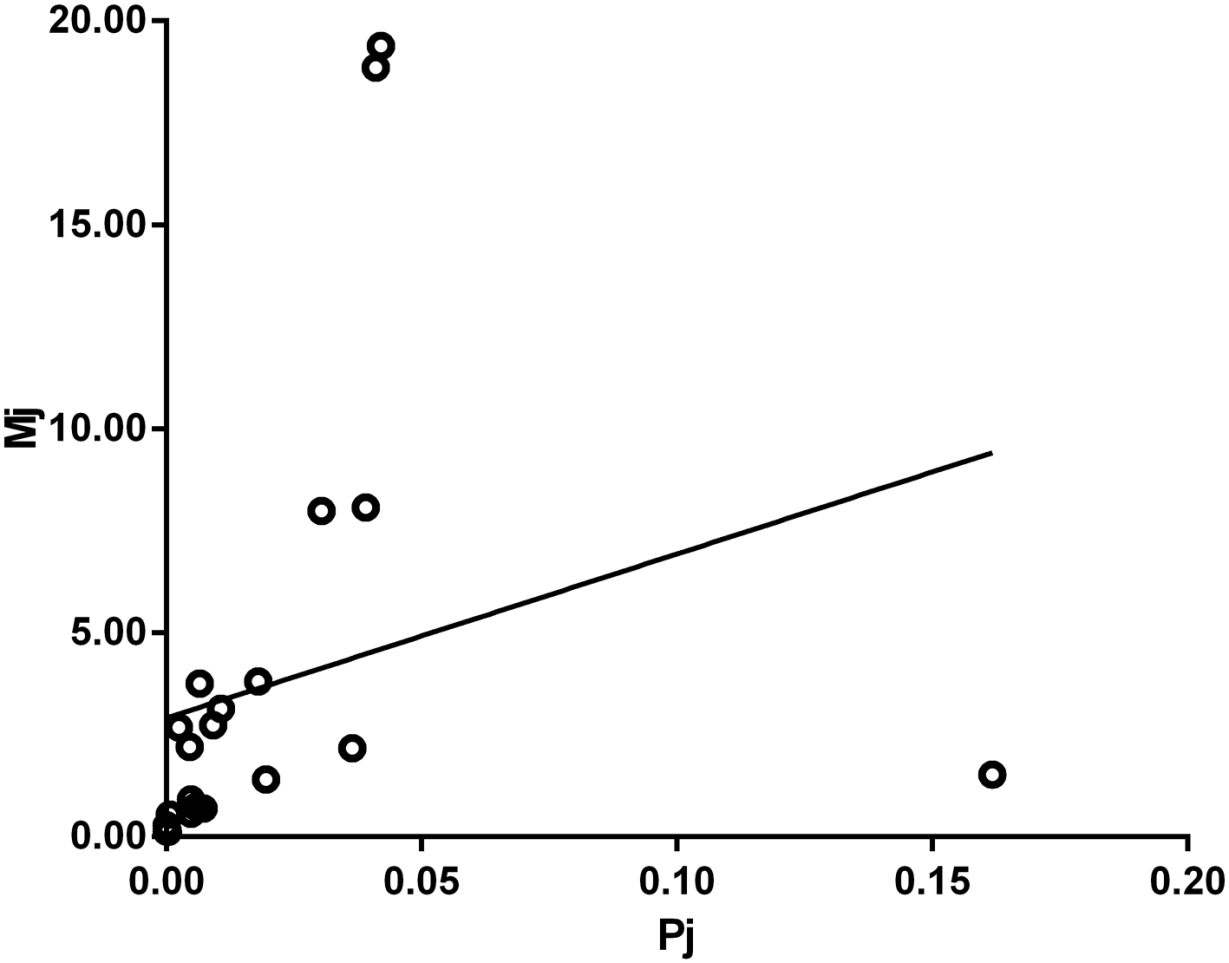
Regression model with all the 22 subpopulations of known size.

Furthermore, as shown in Fig 1, only 6.6% variability (R^2^ = 0.066) could be explained through the regression model with all the 22 subpopulations. Therefore, we applied a graphical analysis of residuals and eliminated 9 subpopulations, which were less fitting the linear relationship between subpopulation sizes and mean number recalled by participants, and the R^2^ increased to 0.814.

**Fig 2:**
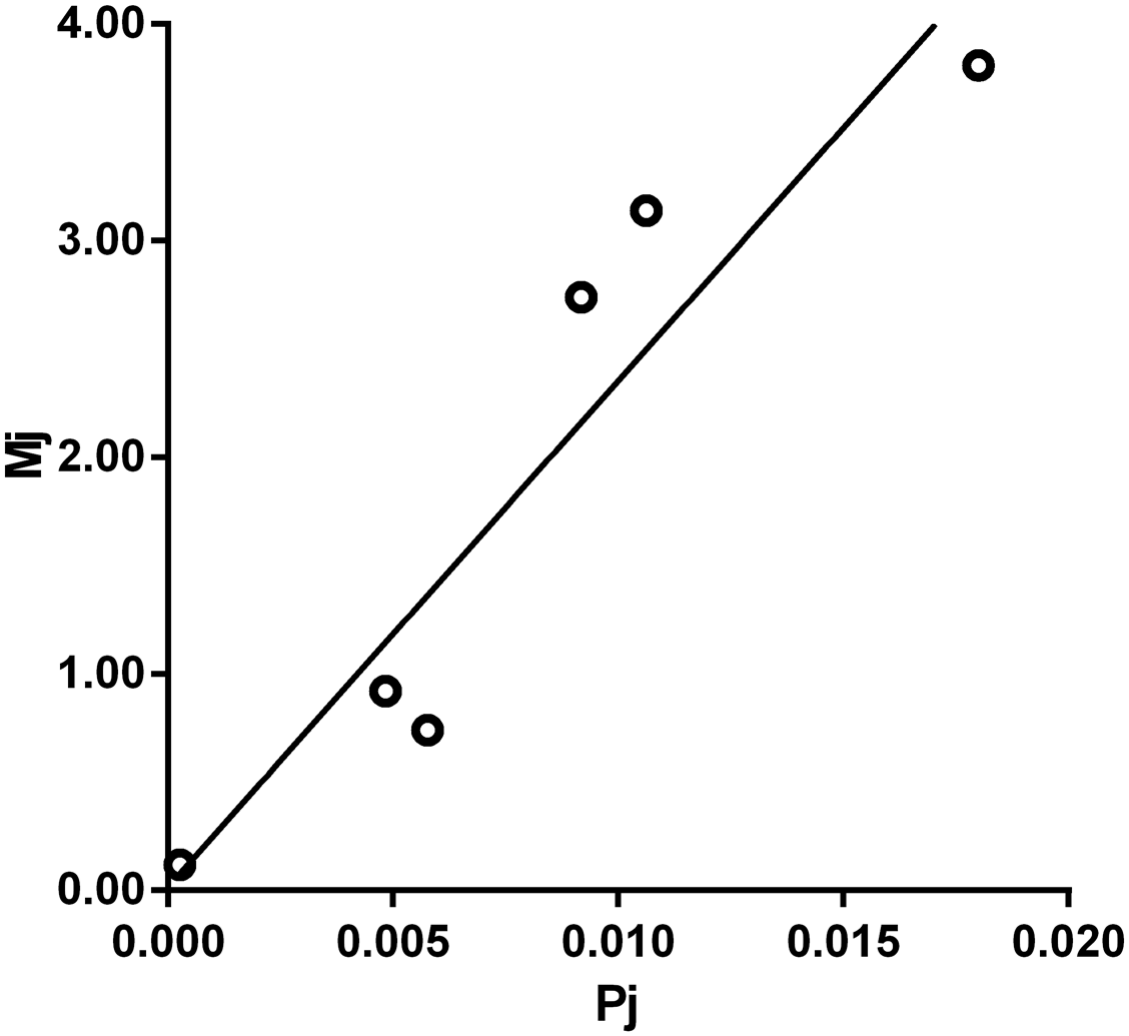
Regression model with the included 6 subpopulations of known size.

Combined the graphical analysis of residuals with the backward estimation method, we eliminated 16 subpopulations in total (Table 3). The subpopulations applied in the adjustment of *c* value were as follows: junior high school students in 2013, men who married in 2013, men who divorced in 2013, people who died in 2013, people who went to jail in 2013, and people who participated in commercial insurance in 2013. With the adjustment of mentioned subpopulations, the average social network size (*c* value) was 236 (95% CI: 224–247). After adjustment, the R^2^ substantially increased to 0.880 (Fig 2).

### Estimation and adjustment of MSM population size

Based on the formula (3), the crude estimate of the MSM population size was 18881 (95% CI: 14800–22971). To minimize the “transmission effect”, which could cause potential underestimation of MSM population size, we made an adjustment with social respect towards MSM. The respect level for MSM were coded using a scale of 1 (very low disagree) to 5 (very high). In the present study, there were 622 (15.9%) participants admitted that they knew MSM. The average number of acquaintances in MSM was negatively associated with participants’ respect level towards MSM (Spearman R = 0.351, P<0.001) (Table 4). According to the average number of acquaintances in MSM and their corresponding respect level, a social respect factor was calculated to be 1.925. After adjustment for the social respect factor, the MSM population size was estimated to be 36354 (95% CI: 28489–44219), which accounted for approximately 0.28% of the target male adult population in Shanghai.

**Table 4:** The average estimate number of acquaintances in MSM by respect level.

Respect level	Sampling size	The average number of acquaintances in MSM	Weight coefficient in each level
Very high	147	1.04	6.50
High	784	0.21	1.31
General	1596	0.16	1.00
Low	903	0.09	0.56
Very low	475	0.04	0.25
Mean weight coefficient	—	—	1.925

## Discussion

To plan and prioritize health interventions for high-risk populations, it is important to know the size of the target populations. The current study focused on HIV/AIDS high-risk populations and estimated the size of MSM populations in Shanghai, China, to be 36354 with a plausible range of 28489–44219 by using the NSUM. To date, two studies have used the NSUM to estimate the MSM population size including one from China [25]. Our results are comparable to the previous study in Chongqing, China, which demonstrated similar prevalence of MSM among male population [24]. The Chongqing study used the same network scale-up method as ours; it is also one of 4 major metropolitan cities in China and should sharing similar economic and social situation. Our study could provide updated information which are of high priority in the HIV/AIDS prevention and control in this subpopulation, and could benefit the HIV/AIDS prevention for MSM population.

The value of *c* is an important parameter to estimate the size of MSM when using the NSUM. To improve the accuracy of *c* value estimation, various approaches had been used previously. Ezoe et al. applied backward estimation method for the *c* value adjustment [26], while residual plot was used for the adjustment in another study [23]. Since choosing appropriate subpopulations is of paramount importance in the NSUM, we combined backward estimation method and regression model to select subpopulations for final estimation, which excluded 16 subpopulations and yielded a substantial improvement of R^2^. In this particular regards, the present study suggests a practical method, which could be applied in the concrete application of the NSUM for further high-risk population size estimation.

Our study used the method of “known population” and its corresponding adjustment approaches, which produced an average social network size (*c*) of 236 which was comparable to results from some previous studies. For example, a study conducted in the U.S. used a similar method and yielded the *c* value of 290 [27], and a study from Japan yielded the *c* value of 206 in urban areas and 197 in rural areas [28]. Noticeably, different methods may produce heterogeneous estimations of *c* values. A study applied six different methods produced six diverse *c* estimates ranging from 97 to 399 [21]. The method of “known population” based on maximum likelihood estimation used in our study is believed to be more accurate than others, and has been applied in a number of studies [21, 29, 30].

In the present study, the population size of MSM aged 18–60 years in Shanghai was estimated to be 36354, which accounted for 0.28% of the target male population. Previous studies estimated the population size of MSM by using different methods and had substantial variations. A study conducted in Shanghai documented that 6.6–7.1% of adult males aged 15–49 years were MSM [31], while another study indicated that MSM accounted for 1.0% and 0.3% of adult population in Beijing and Harbin [32]. A study in Japan estimated a MSM proportion of 2.87% among the total male population [26]. These discrepancies are likely resulted from estimation methods used or geographic dissimilarities or both. Of note, we are also surprised with the discrepancy between the present study and earlier estimates in Shanghai. It may be due to different method applied. Moreover, previous studies using multiplier methods for MSM size estimation have consistently shown that prevalence of urban MSM elsewhere in China were much lower than the earlier estimates in Shanghai [33]. We speculate that there was an overestimate of MSM population in the previous study in Shanghai, which needs to be explored by further investigation. Generally, the NSUM has used different adjustment approaches. Some used backward estimation, while others applied regression model [26, 34]. In the present study, we combined regression model and backward estimation for the selection of subpopulation selection, which could to be more robust.

Traditional approaches such as multiply and capture-recapture require contacting the high-risk populations directly. The method applied in the present study took steps toward reducing some problems in the traditional methods including capture-recapture that requires distinct samplings of the population [35] and the anonymity of subjects in the matching process [36]. However, previous research shown that, different methods such as multiplier and capture-recapture method have their own advantages and disadvantages in the estimation of hard-to-reach populations [37]. To make comparison between applications of these methods, we have retrieved literatures on the application of other methods in MSM size estimation. Such as Wang Cheng [38] used the method of capture-mark-recapture to estimate the size of MSM in Guangdong Province, the result was comparable to our study. Wang Liyan [32] used multiplier method to estimate the size of MSM in Beijing and Harbin. Our result were closer to Harbin city but below Beijing city. And the study of Guo W [25] used the method of NSUM which produce the similar result to ours. Based on these results, we may identify some similarities by different methods. In the future high risk population size estimation, a combination of varies methods are highly warranted to provide corroboration.

There are some limitations for the current study. First, we did not take the sample design into consideration when performing variance estimation in the present study. Second, there are innate biases of network scale-up method of barrier effect, transmission effect and estimation effect [20]. Third, our study used combination of regression model and backward estimation to exclude unsuitable subpopulations to adjust for the initial value of *c*, which needs a further appraisal for the accuracy. Finally, since the respect adjustment in the study itself is an estimate. Until now, no reliable adjustment approach has been accepted unanimously for dealing with this uncertainty. It is clearly an interesting issue, which requires a further investigation.

In conclusion, we used the network scale-up method to estimate the MSM population size in Shanghai, and this method can be used for the estimation of other high-risk and hard-to-reach populations. Timely and accurate estimation of these populations is urgently needed for local governments to effectively plan health interventions and resource allocations.
